# Improved Laser Damage Threshold of In_2_Se_3_ Saturable Absorber by PVD for High-Power Mode-Locked Er-Doped Fiber Laser

**DOI:** 10.3390/nano9091216

**Published:** 2019-08-28

**Authors:** Xile Han, Huanian Zhang, Shouzhen Jiang, Chao Zhang, Dengwang Li, Quanxin Guo, Jinjuan Gao, Baoyuan Man

**Affiliations:** 1 Shandong Provincial Key Laboratory of Optics and Photonic Device, School of Physics and Electronics, Shandong Normal University, Jinan 250014, China; 2 Institute of Data Science and Technology, Shandong Normal University, Jinan 250014, China; 3 Shandong Province Key Laboratory of Medical Physics and Image Processing Technology, School of Physics and Electronics, Shandong Normal University, Jinan 250014, China; 4 Collaborative Innovation Center of Light Manipulations and Applications in Universities of Shandong, Shandong Normal University, Jinan 250014, China

**Keywords:** nonlinear optical materials, indium selenide, physical vapor deposition, mode-locked fiber lasers, saturable absorber

## Abstract

In this study, a double-end pumped high-power passively mode-locked erbium-doped fiber laser (EDFL) was realized by employing a few-layered In_2_Se_3_ flakes as a saturable absorber (SA). Herein, the uniform large-scale In_2_Se_3_ flakes were synthesized by the physical vapor deposition (PVD) method. The PVD-In_2_Se_3_ SA exhibited a remarkable damage threshold of higher than 24 mJ/cm^2^. Meanwhile, the PVD-In_2_Se_3_ SA had a modulation depth and saturable intensity of 18.75% and 6.8 MW/cm^2^, respectively. Based on the In_2_Se_3_ SA, the stable bright pulses emitting at 1559.4 nm with an average output power/pulse energy/pulse duration of 122.4 mW/5.8 nJ/14.4 ns were obtained successfully. To our knowledge, 122.4 mW was the new major breakthrough of mode-locked Er-doped fiber lasers. In addition, this is the first demonstration of the dark-bright pulse pair generation based on In_2_Se_3_ SA. The maximum average output power of the dark-bright pulse reached 121.2 mW, which also showed significant enhancement in comparison with previous works. Our excellent experiment results fully prove the superiority of our experimental design scheme and indicate that the PVD-In_2_Se_3_ could operate as a promising highly-nonlinear photonic material for a high-power fiber laser.

## 1. Introduction

Ultrafast fiber lasers have wide applications in a wide variety of areas, such as information transmission, frequency metrology, scientific research and so on. Over the past few decades, ultrashort pulses fiber lasers have received much attention due to their potential applications in industrial manufacturing, environmental monitoring, toxic gas detection, biomedical, defense, optical sensing and optical imaging. Currently, several passively mode-locked techniques have been employed for generating pulsed laser operations. In 2016, Ivanenko et al. reported a mode-locked long fiber master oscillator based on a nonlinear amplifying loop mirror (NALM) with intra-cavity power management, and achieved a record-high pulse energy exceeding 12 μJ [1]. In addition, various types of saturable absorbers (SAs), including semiconductor saturable absorber mirrors (SESAMs) [2,3], single-wall carbon nanotubes (SWCNTs) [4,5,6], and graphene [7,8,9], have been considered the most promising approach to obtain mode-locked pulses. As known, SESAM has been widely employed in a majority of commercial mode-locked fiber lasers to achieve ultrashort pulses. However, SESAM shows some drawbacks, such as a narrow absorption bandwidth, low damage threshold, complex fabrication and packaging and high cost, which have restricted its further advanced development. In addition, SWCNTs have also been widely used as SAs due to their ultrafast excited state carrier dynamic, high damage threshold and low saturation threshold. However, the absorption bandwidth of SWCNTs depends highly on its diameter, which restricts its practical applications. Moreover, graphene is applicable as a broadband SA due to its Dirac-like electronic band structure. In 2009, Zhang et al. reported on a graphene-based erbium-doped mode-locked fiber laser [9]. Since then, a variety of atomically thin layered materials have been extensively investigated and employed as the SA to obtain mode-locked pulses. Transition metal dichalcogenides (TMDs) [10,11,12,13,14] emerged as a novel kind of layered nanomaterial exhibiting the unique layer-number dependent bandgap properties, which have shown a great potential in nanoelectronics, optical sensing and optoelectronics [15]. However, the limited carrier mobility in the monolayer restricts its advanced application. Recently, topological insulators (TIs) (Bi_2_Se_3_, Bi_2_Te_3_, Sb_2_Te_3_) [16,17,18,19,20] with larger modulation depths and high optical nonlinearity are attracting great interest in laser photonics. In 2012, Bernad et al. first demonstrated the saturable absorption of Bi_2_Te_3_ [20]. Black phosphorus (BP) [21,22] is beneficial to its application in the field of near infrared optoelectronic materials due to its adjustable direct band gap. Regretfully, broadband SAs were still subject to a low laser damage threshold in practical applications.

Recently, indium selenide (In_2_Se_3_), a layered semiconducting chalcogenide, has attracted extensive attention for its various exciting physics properties. In_2_Se_3_ is considered to be a promising material for the applications in photo-voltaic devices [23], phase change memory [24], and especially in opto-electronics [25]. Previous research indicated that In_2_Se_3_ was also available in at least five different phases and crystal structures (α, β, γ, δ, and κ) existing at various temperatures [26]. It is composed of vertically stacked Se-In-Se-In-Se quintuple layers, held together through weak van der Waals forces [27]. Moreover, In_2_Se_3_ shows a tunable thickness-dependent optical bandgap ranging from 1.45 to 2.8 eV [28]. Meanwhile, In_2_Se_3_ as a direct band gap III−VI semiconductor, possesses ultrahigh photodetection responsivity with a fast response time [29]. Therefore, In_2_Se_3_ is highly suited to ptoelectronic devices and photonics applications. In the past several decades, the related properties of α-In_2_Se_3_ have been extensively studied. However, its nonlinear optical properties have rarely been investigated and are rarely used as SAs in a passively mode-locked operation.

At present, a few-layered In_2_Se_3_ flakes have been successfully prepared by several methods, mainly including mechanical exfoliation (ME) [29], chemical vapor deposition (CVD) [27], and physical vapor deposition (PVD) [30]. However, a few-layered In_2_Se_3_ flakes obtained with the ME method led to an uncontrollable size and random thickness, which limits the nonlinear optical response of In_2_Se_3_. Compared with the ME method, the thickness of In_2_Se_3_ flakes that prepared by CVD and PVD methods could be controlled accurately. Moreover, the PVD method could reduce defects of the In_2_Se_3_ flakes and achieve highly uniform films with large areas, which could improve nonlinear optical properties. Additionally, the In_2_Se_3_ flakes prepared by the PVD method possess a higher crystal quality, which could exhibit excellent laser damage threshold.

Recently, for optoelectronic devices, Zhou et al. demonstrated that the atomically layered In_2_Se_3_ exhibit p-type semiconducting behaviors with the mobility up to 2.5 cm^2^/Vs [31]. Meanwhile, for the ultrafast photonic applications, by inserting α-In_2_Se_3_ SA prepared with magnetron-sputtering deposition method into erbium-doped fiber laser (EDFL) systems, Yan et al. obtained soliton pulse with maximal average power/single pulse energy/pulse duration for EDFL of 83.2 mW/2.03 nJ/276 fs, respectively [32]. However, there were no reports on a few-layered In_2_Se_3_ flakes with a large-area prepared by PVD method as SA for high-power mode-locked operations. It is well known that the damage threshold of SA materials is an important parameter to decide whether the material can achieve a high-power laser output and be used in the practice of the material. In general, polymer based SAs have been widely integrated into fiber laser systems. However, high peak power in the cavity may lead to changes in the property of polymer and even damage the SAs.

In this report, a double-end pumped mode-locked EDFL based on In_2_Se_3_ SA for high-power laser output is presented. The uniform large-area atomically thin In_2_Se_3_ flakes were synthesized on fluorophlogopite mica (FM) by the PVD method. In addition, by a transfer process, a few-layered In_2_Se_3_ flakes were directly transferred on the facet of the fiber. This work studied the laser damage threshold of the PVD-In_2_Se_3_ SA, which possessed excellent damage threshold. As is known, the laser damage threshold of SESAM (BATOP, SA-1550-35-2ps-x) is 1.5 × 10^3^ μJ/cm^2^. Compared with the SESAM, the laser damage threshold of the In_2_Se_3_-FM SA reached as high as 24 mJ/cm^2^. Meanwhile, the nonlinear optical properties of In_2_Se_3_ SA were investigated. It exhibited excellent nonlinear optical performances, such as a large modulation depth (18.75%) and lower saturable intensity (6.8 MW/cm^2^). By employing the In_2_Se_3_ SA in a bidirectional pumping high-power mode-locked EDFL system, a variety of stable mode-locked pulses were obtained. The average output power was 122.4 mW, corresponding to a single pulse energy of 5.8 nJ. The experiment results fully prove that In_2_Se_3_ could be a potentially excellent SA for a high-power mode-locked fiber laser application in practice.

## 2. Preparation and Characterization of the In_2_Se_3_ SA 

### 2.1. Synthesis and Characterization of In_2_Se_3_ Flakes

**Figure 1:**
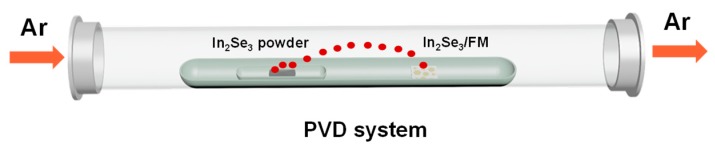
Schematic diagram of the physical vapor deposition for In_2_Se_3_ flakes growth.

In our experiment, the commonly-reported PVD method was employed for preparing high-quality In_2_Se_3_ flakes [30]. The growing progress is illustrated in Figure 1. The In_2_Se_3_ flakes were grown on monolayer FM substrates via van der Waals epitaxy in a horizontal tube furnace (OTL1200). The In_2_Se_3_ power (99.99%, Alfa Aesar, Beijing, China) as an evaporation source was placed at the constant-temperature zone of the tube furnace heated to 750 °C for 60 min. The vapor was transported downstream by 50 sccm Ar gas with pressure controlled at 15 Pa. The In_2_Se_3_ flakes were grown on FM substrate placed 12 cm away from the heating center. Finally, the furnace was naturally cooled down to the ambient temperature in the gas flow of Ar.

**Figure 2:**
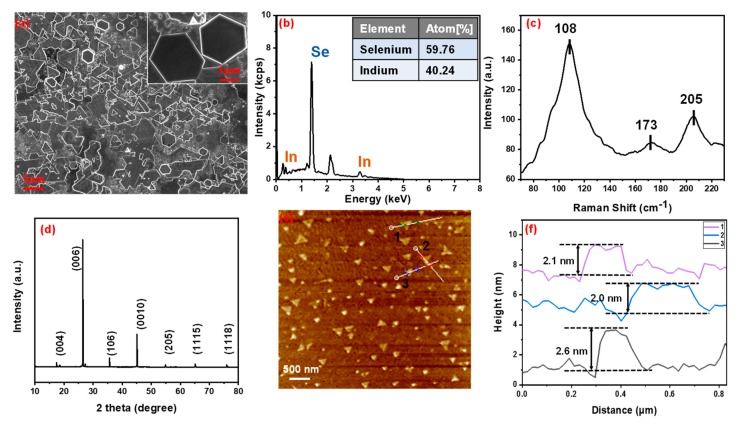
(**a**) SEM image of the In_2_Se_3_ flakes; (**b**) EDS spectroscopy of the In_2_Se_3_ flakes; (**c**) Raman spectrum of the In_2_Se_3_ flakes; (**d**) X-ray diffraction of the In_2_Se_3_ flakes; (**e**) AFM image of In_2_Se_3_ flakes; (**f**) Height profile of In_2_Se_3_ flakes measured along the white line in (e).

Figure 2a shows a typical scanning electrical microscope (SEM) (Sigma 500, ZEISS, Oberkochen, Germany) image of the prepared In_2_Se_3_ flakes on FM. It is obvious that most flakes exhibit asymmetric hexagonal and truncated trigonal morphology shapes, which are along the horizontal direction. Meanwhile, an energy dispersive spectrometer (EDS, XFlash 6130, Bruker, Germany) was employed for investigating the characteristic of the elemental composition. As shown in Figure 2b, the stoichiometric ratio of Se (59.76%) and In (40.24%) is estimated to be 3:2. The structural characterization of the prepared In_2_Se_3_ flakes was also tested by a Raman spectroscopy (Horiba HR Evolution) with excitation light of 532 nm. The test result is displayed in Figure 2c, where the three peaks at ~108, ~173 and ~205 cm^−1^ are attributed to A_1_ (LO + TO), A_1_ (TO), and A_1_ (LO) phonon modes of In_2_Se_3_. These Raman features unequivocally indicate the successful preparation of In_2_Se_3_ flakes [33]. A blue shift in lattice phonon mode of A_1_ (TO) might be due to the oxidized caused by the Raman excitation laser [30]. In addition, the crystal structure of the In_2_Se_3_ flakes was investigated through X-ray diffraction (XRD) (Bruker D8 ADVANCE, Billerica, MA, USA). Figure 2d shows the relatively higher intensity of the (006) peak which indicates that the In_2_Se_3_ flakes exhibit a well-layered structure and high crystallinity. As shown in Figure 2e, atomic force microscopy (AFM, Bruker Multimode 8, Germany) was used to determine the thickness of the In_2_Se_3_ flakes on FM. Figure 2f shows the lateral height profile of the In_2_Se_3_ flakes. The thickness of the marked samples ranged from approximately 2.0 to 2.6 nm.

### 2.2. Preparation and Characterization of In_2_Se_3_ SA

In the experiment, In_2_Se_3_ flakes based on a monolayer FM were peeled off into a few layers by pyrolysis tape. Then, the few layers In_2_Se_3_-FM was directly attached onto a fiber end-facet for preparing an all fiber SA with a high laser damage threshold.

**Figure 3:**
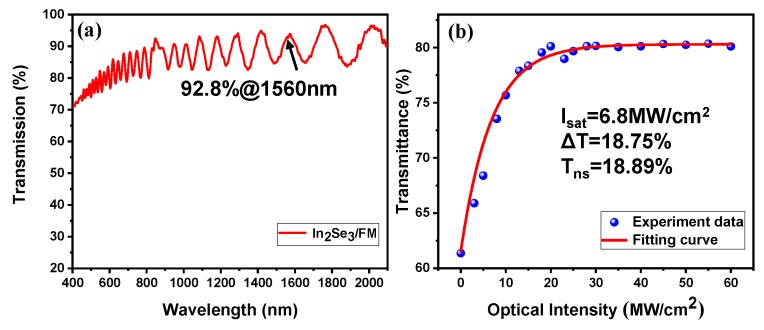
(**a**) Linear transmission of the In_2_Se_3_-FM saturable absorber (SA) versus the wavelength. (**b**) The nonlinear absorption property of the In_2_Se_3_-FM SA.

The linear transmission of In_2_Se_3_-FM from 400 to 2000 nm was measured by using a UV/vis/NIR spectrophotometer (Hitachi U-4100). As shown in Figure 3a, In_2_Se_3_-FM SA exhibits a high transmission of 92.8% at 1560 nm. Here, the linear transmission shows a fringe, which was caused by a spectral interference caused by a thin film of a certain thickness. In addition, the nonlinear absorption properties of the In_2_Se_3_-FM SA were investigated by a power-dependent transmission technique. The representative result is shown in Figure 3b. The data for the transmission is fitted by the following equation [34]:

(1) $$T\left(I\right)=1-\Delta T\times \exp\left(-I/I_{sat}\right)-T_{ns}$$

where *T* is the transmission, Δ*T* is the modulation depth, *I* is the input intensity of the laser, *I_sat_* is the saturation intensity, *T_ns_* is the non-saturable loss. According to the experimental results of fitting, the saturation intensity, modulation depth and non-saturable loss is approximately 6.8 MW/cm^2^, 18.75% and 18.89%, respectively.

## 3. Experimental Setup

**Figure 4:**
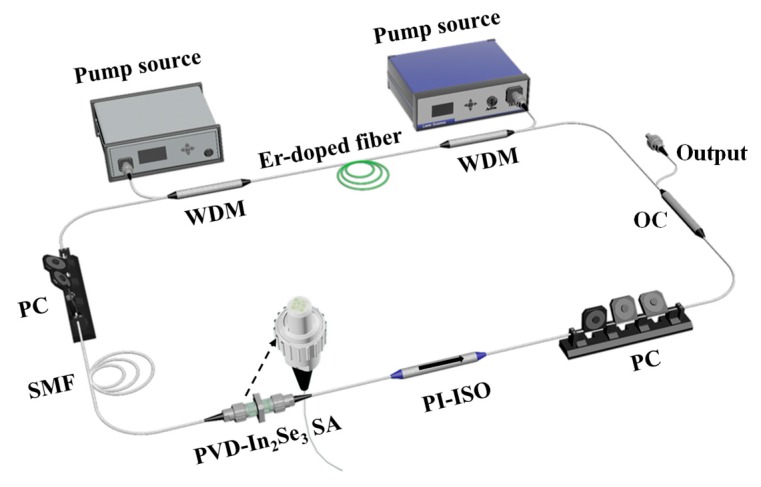
Schematic illustration of the typical all-fiber ring cavity of the mode-locked erbium-doped fiber laser (EDFL) based on the PVD-In_2_Se_3_ SA.

The experimental setup of the double-end pumped fiber laser was shown in Figure 4. As is shown, a piece of 62 cm high-concentration erbium-doped fiber (EDF, LIEKKI Er-80-8/125) with a group velocity dispersion (GVD) of −19.5 ps^2^/km was used as the gain medium. Two 976 nm laser diodes (LDs) were employed as the pump source. Two 980/1550 nm wavelength division multiplexers (WDMs) were used to couple the pump power into the ring laser cavity. A polarization-insensitive isolator (PI-ISO) was employed to ensure the unidirectional laser operation. Two polarization controllers (PCs) were employed to adjust the cavity polarization and intra-cavity birefringence. A 40/60 optical coupler was used to extract a 60% lasing signal for monitoring. A piece of 110 m single-mode fiber (SMF-28e) with a dispersion parameter of 17 ps/nm/km was added into the cavity for dispersion management. The total length of the cavity was approximately 120 m. Thus, the total net cavity dispersion was calculated to be −2.6 ps^2^. The performance of the output laser was record by a fastspeed photodetector (3G), a digital oscilloscope (DPO4054), an optical power meter (BAGGER D50A), an optical spectrum analyzer (AQ6317B) and a radio-frequency (RF) spectrum analyzer (R&S, PC1000, 1GHz).

## 4. Experimental Results and Discussions

A ~120 m long laser cavity was designed for obtaining mode-locked operations. In addition, the phenomena of self-mode-locked or Q-switched operations were always recorded within a long-length ring fiber laser cavity due to the Kerr effect under high pump power. Thus, firstly, a piece of FM substrate was inserted into the ring laser cavity instead of the In_2_Se_3_ SA for testing the possibility of self-mode-locked or Q-switched operations. By adjusting the pump power and the polarization states of PCs, neither the self-mode-locked nor Q-switched pulse generations were detected, which excluded the Kerr effect and the saturable absorption effect of the FM substrate. Then, the In_2_Se_3_-FM SA was inserted into the ring cavity, and self-started mode-locked operations were achieved when the pump power reached 313 mW. The fiber laser exhibited a high lasing threshold due to a large output coupling ratio and a relatively large insert loss of the SA. In our experiment, the stable mode-locked operations can be maintained with the pump power increasing from 313 to 1324 mW. As is known, the formations of different solitons were due to the balancement between the various nonlinear optical effects, the total laser gain and loss and the net dispersion value within the laser cavity. In our work, by adjusting the pump power and the states of the PCs, the different soliton operations were recorded successfully, which is discussed in detail below.

### 4.1. Bright Pulses

**Figure 5:**
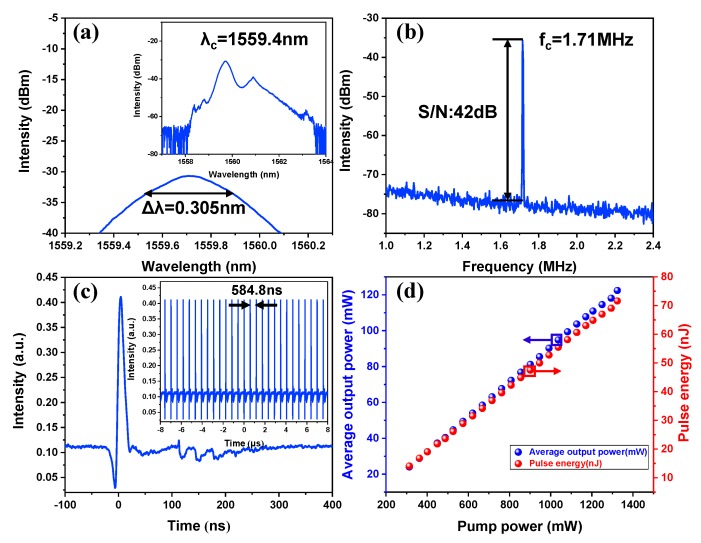
Typical optical characteristics of the bright pulse at the pump power of 1324 mW. (**a**) Optical spectrum with indicated 3dB bandwidth. Inset: The output optical spectrum; (**b**) radio-frequency (RF) spectrum at a fundamental frequency of 1.71 MHz with 300 Hz resolution; (**c**) the corresponding single pulse profile of the bright pulse and the inset shows the typical pulse train; (**d**) average output power versus the pump power.

Firstly, the wide-reported bright pulses were obtained in our work easily. The mode-locked characteristics of the bright pulse under the pump power of 1324 mW were depicted in Figure 5. In detail, Figure 5a shows the optical emission spectrum of the bright pulse, which centered at 1559.4 nm with a 3 dB spectral bandwidth of 0.305 nm. However, no Kelly sidebands were observed in the spectrum, which indicate the EDFL was not operated in the conventional soliton regime. The radio frequency (RF) spectrum was measured and shown in Figure 5b. The signal-to-noise ratio (SNR) at the fundamental repetition rate of 1.71 MHz is approximately 42 dB. In order to avoid the photodetector and optical spectrum analyzer from being damaged at high output power, the output pulse was split by two output couplers with coupling rates of 40/60 and 50/50, respectively, which reduced the SNR. Accordingly, the SNR should be greater than 42 dB. Meanwhile, it also limited the wideband RF output. Figure 5c shows an oscilloscope trace of the obtained single pulse. The inset shows a typical pulse train of the mode-locked operation with a pulse-to-pulse interval of 584.8 ns, corresponding to a fundamental repetition rate of 1.71 MHz, which matches well with the total cavity length of 120 m. As is shown, the full width at half-maximum (FWHM) of the bright pulse is 14.4 ns. It is generally known that the dispersion has an obvious effect on broadening the width of the pulse in an anomalous dispersion regime. Thus, the wide pulse width is mainly caused by the large net dispersion value. However, due to the limitation of response time of detector and oscilloscope, the actual pulse width will be less than 14.4 ns. Regrettably, due to the lack of autocorrelator, the actual pulse width was not measured. The average output power as a function of pump power is recorded in Figure 5d. The maximum average output power is 122.4 mW at the pump power of 1324 mW. However, the single pulse energy is limited to 5.8 nJ due to the direct current power between the pulses. In our experiment, no material damage was found, which indicates that the laser damage threshold of the PVD-In_2_Se_3_ SA should be greater than 24 mJ/cm^2^. In addition, in further works, we hope to achieve large-energy mode-locked operations by adjusting the laser parameters and further optimizing the preparation of In_2_Se_3_ flakes.

To fully prove the advantages of the PVD-In_2_Se_3_ based EDFL, the results of a series of high-power mode-locked fiber lasers based on different SAs are summarized in Table 1. It is clear that the highest average output power based on mode-locked EDFL was obtained in our work due to the combination of the high pump source and the high damage-threshold SA. It is worth noting that most of the work exhibits the pulse duration of picoseconds or even sub-picoseconds, which has broad application prospects in biomedical, nonlinear optics and ultrafast optics. However, its output power is limited to tens of milliwatts [32,36,48]. When a picosecond or femtosecond pulse is amplified, the high peak power results in a strong nonlinear effect, which is not conducive to the application of high-power lasers. Compared with picosecond or femtosecond mode-locked pulses, nanosecond mode-locked pulses have advantages such as a strong chirp, large pulse width, low peak power, and small nonlinear phase shift accumulation. Therefore, the nanosecond fiber lasers with high pulse energy can be used directly as seed sources to achieve higher power output through single or multi-stage amplification systems. Our results indicate that the PVD-In_2_Se_3_-FM based mode-locked fiber laser can be used as a seed source for a chirped pulse amplification (CPA) system.

### 4.2. Dark-Bright Pulse Pairs

**Figure 6:**
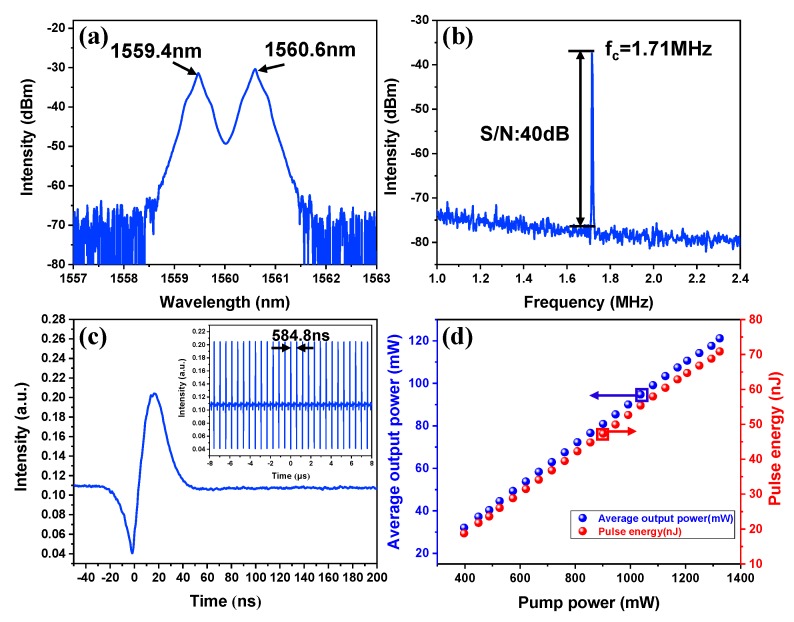
Typical optical characteristics of dark-bright pulse pairs at the pump power of 1324 mW. (**a**) The output optical spectrum; (**b**) RF spectrum at a fundamental frequency of 1.71 MHz with 300 Hz resolution; (**c**) the corresponding single pulse profile of the dark-bright pulse pair and the inset shows the pulse train; (**d**) average output power versus the pump power.

In the experiment, just by adjusting the states of the PCs, stable dark-bright pulse pair mode-locked generation has also been detected. Here, the optical characteristics of the dark-bright pulse pair under the pump power of 1324 mW were discussed. The typical output optical emission spectrum is recorded in Figure 6a. It is noteworthy that the spectrum shows a typical M-shape profile with a dual-wavelength centered at 1559.4 and 1560.6 nm, respectively, which is similar to the previous report [18]. As described above, the large-area In_2_Se_3_ flakes synthesized by the PVD method exhibit a large nonlinear optical effect due to their high crystal quality and high flatness. Therefore, the high nonlinear optical effect within the laser cavity facilitates the generation of multi-wavelength mode-locked pulses. Figure 6b shows the RF spectrum of the laser at a fundamental repetition rate of 1.71 MHz. The SNR exceeded 40 dB, which indicates a high stability of the dark-bright pulse pair mode-locked operation. Figure 6c shows the corresponding single pulse profile of the dark-bright pulse pair. However, compared with bright pulse, the dark pulse exhibits different pulse intensity and pulse width, which is different from the previous study [50]. In our opinion, this is caused by the high-order nonlinear effect of the In_2_Se_3_ SA. Meanwhile, the inset shows the typical dark-bright pulse pair train with a period of 584.8 ns, corresponding to a fundamental repetition rate of 1.71 MHz, which verifies the mode-locked operation of the fiber laser. In addition, by adjusting the pump power and the PCs, the dark-bright pulse pair emissions also could be obtained. The relationship between the pump power and average output power is recorded in Figure 6d. The mode-locked threshold of the dark-bright pulse pair operation was 397 mW, which was higher than the bright pulse mode-locked operation. The generation of dark-light pulse pair may be the result of the nonlinear refractive index of In_2_Se_3_ flakes interacting with the high nonlinear effects caused by the higher power in the laser cavity [51]. The average output power and the single pulse energy are 121.2 mW and 2.7 nJ, respectively. To the best of our knowledge, this is the highest output power of dark-bright pulse pair mode-locked operations based on EDFL.

**Figure 7:**
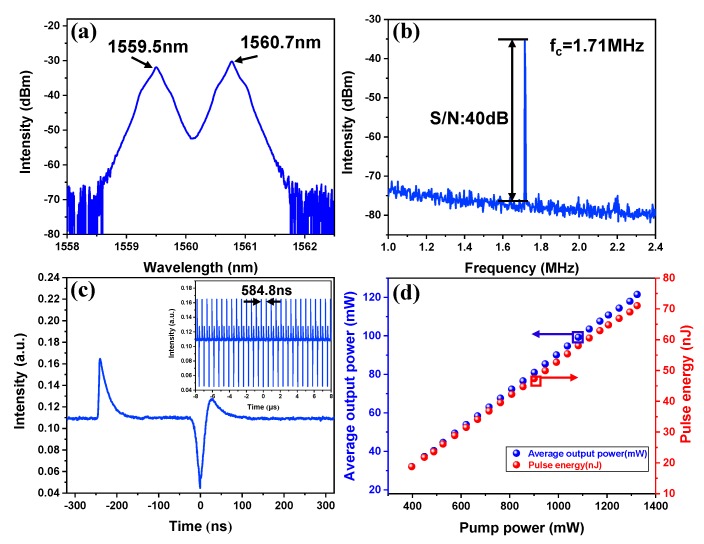
Typical optical characteristics of bright-dark pulse pair at the pump power of 1324 mW. (**a**) The output optical spectrum; (**b**) RF spectrum at a fundamental frequency of 1.71 MHz with 300 Hz resolution; (**c**) the corresponding single pulse profile of the bright-dark pulse pair and the inset shows the typical pulse train; (**d**) average output power versus the pump power.

Accordingly, the adjustment of the polarization state of the PCs also led to the formation of stable bright-dark pulse pair mode-locked operations. Figure 7 shows the output characteristics of the bright-dark pulse pair at the maximum pump power. It is obvious that the bright and dark pulses are separated from each other. Figure 7a shows the typical emission optical spectrum with a dual-wavelength centered at 1559.5 and 1560.7 nm, respectively. For investigating the stability of the bright-dark pulse pair mode-locked operation, its RF spectrum was measured. As shown in Figure 7b, the fundamental frequency is also located at 1.71 MHz with a SNR over 40 dB, indicating that the bright-dark pulse pair operates in a relatively stable regime. Figure 7c shows the corresponding single pulse profile of the bright-dark pulse pair. Clearly, the bright and dark pulse can be observed simultaneously, which the pulse interval exceeds to approximately 200 ns. Figure 7d depicted the relationship between the average output power versus the pump power. As is shown, when the pump power increases from 397 to 1324 mW, the average output power grows from 32.1 to 121.5 mW, corresponding to a maximum single pulse energy of 2.8 nJ. This is the first demonstration based on In_2_Se_3_ SA in a bright-dark pulse pair mode-locked EDFL. In addition, there were no bright pulse and bright-dark pulse pairs observed no matter how adjusted the cavity polarization state was when the In_2_Se_3_ SA was removed.

## 5. Conclusions

In conclusion, this study has demonstrated PVD-grown large-area In_2_Se_3_ flakes as SA for generating high-power and a large-energy passively mode-locked EDFL. The In_2_Se_3_-FM SA exhibited a high laser damage threshold of higher than 24 mJ/cm^2^, a large modulation depth of 18.75% and a saturable intensity of 6.8 MW/cm^2^. Based on the In_2_Se_3_-FM SA, the stable mode-locked pulse with a maximum average output power and a single pulse energy of 122.4 mW and 5.8 nJ were obtained successfully. To our knowledge, this is the highest output power achieved in a mode-locked EDFL based on two-dimensional (2D) materials. In addition, the dark-bright pulse pair operations with recorded high output powers were also observed for the first time. Thus, our experimental results with obvious enhancements in comparison with previous works fully indicate the superiority of our experiment design and is expected to provide an absolutely new reference for generating high-power mode-locked fiber lasers based on 2D materials as SAs.

## Figures and Tables

**Table 1:** Comparison of 1.5 μm high-power mode-locked fiber lasers based on different SAs ^a^.

SA	Fabrication	*λ_c_* nm	*f_c_* MHz	*τ* ps	*P*_ave_ mW	*E*_pulse_ nJ	Ref.
**Graphene**	ME	1568	16.34	0.844	30	1.84	[35]
**Graphene**	RGO	1555	15.36	0.51	80	5.2	[36]
**Bi_2_Se_3_**	SPE	1562.1	3.54	25.16 ns	10	2.824	[37]
**Bi_2_Se_3_**	LPE	1560.5	0.5376	—	33.8	62.87	[17]
**Bi_2_Te_3_**	PLD	1564.1	2950	0.92	45.3	0.01536	[38]
**Bi_2_Te_3_**	Solvothermal	1571	10.71	6.2	30	2.8	[39]
**Bi_2_Te_3_**	ME	1560	1.7	12.8 ns	32.9	22.4	[40]
**Sb_2_Te_3_**	PLD	1530	94	—	12	0.127	[41]
**WS_2_**	CVD	1568.3	0.487	1.49	62.5	128.3	[42]
**WS_2_**	LPE	1531.5/1557.5	2.14	11	14.2	6.64	[43]
**WS_2_**	PLD	1561	101.4	0.246	18	—	[44]
**WSe_2_**	CVD	1557.4	63.133	0.1635	28.5	—	[45]
**WSe_2_**	CVD	1562	58.8	0.185	30	—	[46]
**MoSe_2_**	BTS	1557.3	3270	0.751	22.8	0.0067	[47]
**MoTe_2_**	MSD	1559	26.6	0.229	57	2.14	[48]
**ReS_2_**	CVD	1565	318.5	—	12	0.037	[49]
**BP**	LPE	1559.5	8.77	0.94	53	—	[22]
**In_2_Se_3_**	MSD	1565	40.9	0.276	83.2	2.03	[32]
**In_2_Se_3_**	PVD	1559.7	1.71	14.4 ns	122.4	5.8	This work

^a^*λ*_c_, the central wavelength; *f*_c_, the fundamental frequency; τ, the pulse duration; Pave, the output average power; *E*_pulse_, the pulse energy; ME, mechanical exfoliation; RGO, reduced graphene oxide; SPE, solution-phase exfoliation; LPE, liquid-phase exfoliation; PLD, pulsed laser deposition; CVD, chemical vapor deposition; BTS, bath-type sonication; MSD, magnetron-sputtering deposition.
